# Efficacy of toripalimab in combination with anlotinib in recurrent undifferentiated pleomorphic sarcoma of the sinonasal region: a case report with biomarker analysis

**DOI:** 10.3389/fimmu.2025.1541209

**Published:** 2025-05-09

**Authors:** Fang Wu, Junqiao Feng, Hong Wang, Shan Wang, Xiaoguang Cui, Ying Liu, Linli Yan, Kaihong Ye, Rick F. Thorne, Xu Dong Zhang, Ting La

**Affiliations:** ^1^ Department of Oncology, the First Affiliated Hospital of The Fourth Military Medical University, Xi’an, Shaanxi, China; ^2^ National-Local Joint Engineering Research Center of Biodiagnosis & Biotherapy, The Second Affiliated Hospital of Xi’an Jiaotong University, Xi’an, Shaanxi, China; ^3^ Precision Medical Research Institute, the Second Affiliated Hospital of Xi’an Jiaotong University, Xi’an, Shaanxi, China; ^4^ Department of Rheumatology and Immunology, The Second Affiliated Hospital of Xi’an Jiaotong University, Xi’an, Shaanxi, China; ^5^ Department of Radiology, the First Affiliated Hospital of The Fourth Military Medical University, Xi’an, Shaanxi, China; ^6^ Department of Pathology, the First Affiliated Hospital of The Fourth Military Medical University, Xi’an, Shaanxi, China; ^7^ Translational Research Institute of Henan Provincial People’s Hospital and People’s Hospital of Zhengzhou University, Tianjian Laboratory of Advanced Biomedical Sciences, Academy of Medical Sciences, Zhengzhou University, Zhengzhou, Henan, China

**Keywords:** undifferentiated pleomorphic sarcoma, recurrence, toripalimab, anlotinib, case report

## Abstract

**Background:**

Soft tissue sarcoma (STS) typically originates in the muscles and is associated with a poor prognosis. Undifferentiated pleomorphic sarcoma (UPS) is the most commonly diagnosed subtype of STS; however, UPS occurring in the sinonasal region is exceedingly rare and lacks effective treatment options.

**Objective:**

This case report presents a patient with sinonasal UPS who experienced disease progression after surgery and chemotherapy but showed a positive response to combination therapy with toripalimab and anlotinib. Additionally, it explores the underlying biomarkers associated with this case.

**Case:**

A 63-year-old woman with no significant past medical history was diagnosed with sinonasal UPS. The lesions recurred despite seven extensive surgical resections, and standard chemotherapy failed to control the disease, leading to progressive disease (PD).

**Results:**

The patient was treated with a combination of toripalimab and anlotinib, resulting in a significant partial response (PR) after just two cycles. Continued PR was observed after an additional six cycles, indicating the potential for a prolonged response with ongoing therapy. Genotyping and immunohistochemistry revealed that the sarcoma cells were rapidly dividing and enriched in vasculature prior to systemic treatment.

**Conclusion:**

These findings suggest that the combination of toripalimab and anlotinib may be an effective treatment option for advanced cases of UPS in the sinonasal region.

## Introduction

Sinonasal tumors, which originate in the nasal cavities or paranasal sinuses, are rare malignancies with an incidence of fewer than one case per 100,000 individuals annually worldwide (1). Due to the extreme rarity, conducting prospective clinical trials has been unfeasible, and the heterogeneity of this disease has posed significant challenges to developing evidence-based therapeutic strategies (1–3). Mesenchymal-origin sarcomas are derived from supporting tissue and are much rarer than epithelial-origin carcinomas (1). More than 20 histopathological subtypes of sinonasal sarcoma have been reported (4). Among these, undifferentiated pleomorphic sarcoma (UPS) is a group of heterogeneous undifferentiated/unclassified soft tissue sarcoma. At 60 months, the overall and disease-free survival rates for sinonasal sarcoma are 61.3% and 53.2%, respectively, compared to significantly lower rates for UPS at 24% overall survival and 20% disease-free survival (1).

Localized surgical resection is the preferred treatment for primary sarcomas; however, 40%–50% of patients who undergo resection develop distant metastases, with a 5-year survival rate of less than 10% (5, 6). Chemotherapy is the first-line treatment for advanced soft tissue sarcomas (STSs) (7). Anthracycline-based chemotherapeutic regimens such as doxorubicin plus ifosfamide have provided an objective response rate of 29% for UPS (7, 8). Anlotinib is a newly developed, orally multitargeted tyrosine kinase inhibitor (TKI) that suppresses tumor growth and angiogenesis, which has exhibited promising efficacy and manageable toxicity in various cancers (9–11). Anlotinib represents a preferred option as a post-first-line maintenance treatment or as a monotherapy for STS patients who are intolerant to anthracycline chemotherapy (12, 13).

Targeted therapies using immune checkpoint inhibitors (ICIs) to block the binding between programmed cell death-1 (PD-1) and programmed cell death ligand-1 (PD-L1) have achieved good responses in difficult-to-treat malignancies such as lung cancer and melanoma. ICIs are not currently included in the standard treatment protocols for sarcomas, although a number of clinical trials have investigated their benefits in treating different sarcoma types (14). Toripalimab is a recombinant humanized PD-1 monoclonal antibody that has been approved by the US Food and Drug Administration (FDA) for the treatment of adults with metastatic or recurrent, locally advanced nasopharyngeal carcinoma when used with cisplatin and gemcitabine (15, 16). Evidence indicating the efficacy of toripalimab against sinonasal sarcoma is lacking, save for one patient with UPS of the lung showing benefits from the combination treatment of anlotinib and toripalimab therapy (17).

Here, we report our findings involving a patient diagnosed with primary UPS of the sinonasal region whose disease progressed rapidly after endoscopic resection. The aggressive sarcoma relapsed frequently, and after seven surgeries, it became unresectable. No objective responses were obtained with chemotherapy with epirubicin and ifosfamide. Following disease progression, treatment with toripalimab immunotherapy combined with anlotinib provided a partial response (PR) after two treatment cycles without obvious side effects. The patient has benefited from this treatment for 7.5 months.

## Case presentation

**Figure 1:**
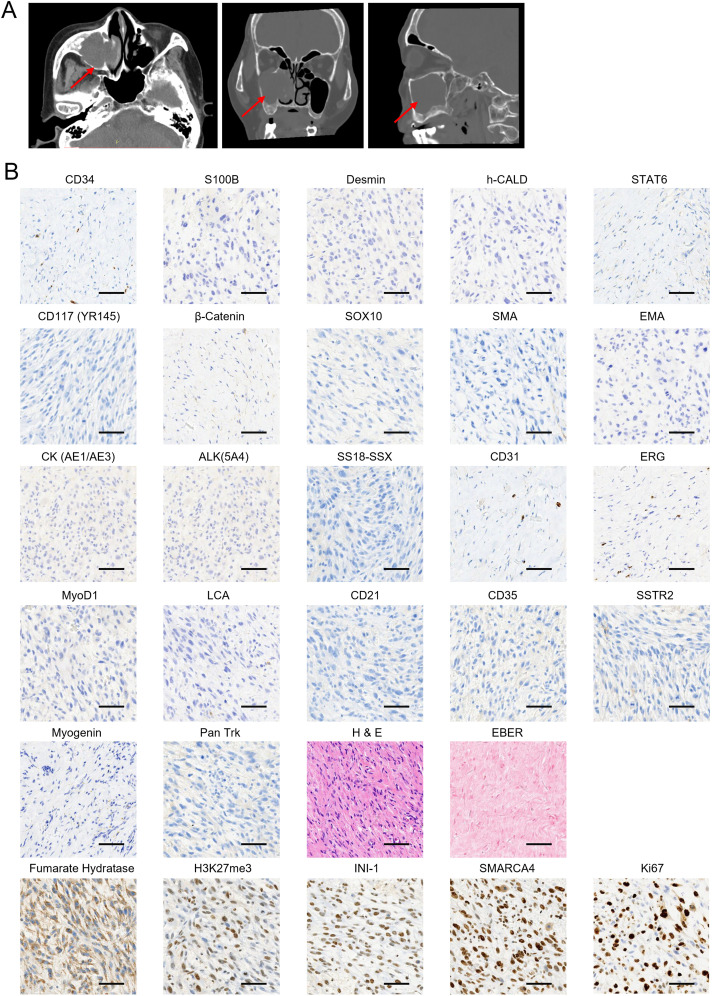
Initial clinical presentation of the sarcoma and biopsy histologic images. **(A)** Horizontal (left), coronal (middle), and sagittal (right) computed tomography (CT) scan of sinus before surgery resection in the local hospital. The tumor is indicated by red arrows. **(B)** A panel of immunohistochemical and *in situ* hybridization markers was performed to confirm the soft tissue sarcoma (STS) subtype diagnosis. Samples were collected on 19 July 2022 during the first surgery. The markers that showed positive staining are displayed in the bottom panel. Scale bar, 50 μm.

In July 2022, a 63-year-old female patient presented to a local hospital with numbness and discomfort on the right side of the face and nosebleeds. A tumor in the paranasal sinus was removed and concluded as mesenchymal sarcoma by biopsy pathology (**Figure 1A**). The patient was then admitted to our hospital for detailed evaluation and treatment. The patient was in fair spirits, displayed normal physical strength and good appetite, and slept well. No significant change in weight was found, and both bowel movements and urination were normal. The patient denied having a significant medical history except for hypertension since 2016, which was well controlled by oral candesartan cilexetil tablets (4 mg/day). Additionally, the patient indicated that there is no family history of any specific health issues. A nasal endoscopy showed postoperative changes in the right nasal cavity. The bilateral inferior turbinate was normal, but the middle turbinate was pale, and the left uncinate process was hypertrophic. The right middle nasal meatus had purulent secretion and was cleaned. The nasal septum had deviated to the left, and the nasopharyngeal mucosa was normal. Sinus magnetic resonance imaging (MRI) indicated that the right maxillary sinus medial wall and middle turbinate were absent after surgery. There was a low-density appearance on the anterior wall and posterior wall of the right maxillary sinus, right orbital inferior wall, right frontal sinus, ethmoid sinus, and maxillary sinus. Fat herniation was noted on the left orbital medial wall.

Further pathological consultation provided a diagnosis of mesenchymal sarcoma, and “endoscopic right maxillary subtotal resection, extended resection of nasal sinus tumors, and partial resection of orbital cardboard” was performed in the Department of Otolaryngology of our hospital on 19 July 2022. Immunohistochemistry (IHC) staining panels were used to confirm the subtypes of STS and guide treatment regimens. The results showed that the sarcoma was negative for CD34, S100 Calcium Binding Protein B (S100B), Desmin, H-Caldesmon Antibody (h-CALD), Signal Transducer And Activator Of Transcription 6 (STAT6), CD117 (YR145), β-Catenin, SRY-Box Transcription Factor 10 (SOX10), smooth muscle Actin (SMA), epithelial membrane antigen (EMA), Cytokeratin AE1/AE3 [CK (AE1/AE3)], Anaplastic lymphoma kinase 5A4 [ALK(5A4)], SS18-SSX, CD31, ERG, MyoD1, leukocyte common antigen (LCA)', CD21, CD35, Somatostatin Receptor 2 (SSTR2), Myogenin, and Pan Trk (**Figure 1B**). In contrast, the staining of fumarate hydratase (Fh), H3K27me3, INI-1, and SWI/SNF Related BAF Chromatin Remodeling Complex Subunit ATPase 4 (SMARCA4) was positive (**Figure 1B**). Tumor cells scored 80% positive for Ki67 staining (**Figure 1B**). *In situ* hybridization (ISH) tests showed the tissue was negative for Epstein–Barr virus (EBV)-encoded small RNAs (EBER) (**Figure 1B**), indicating the absence of EBV infection. The collective IHC staining results indicated that the sarcoma shows a lack of specific differentiation. Furthermore, based on the imaging features observed in the computed tomography (CT) and MRI, clinicians and radiologists diagnosed the soft tissue mass as UPS. Regarding the CT and MRI findings, the lesion originated in the sinus and extended into the orbit, with no involvement of neck lymph nodes or distant metastases. Consequently, the TNM staging was classified as T3N0M0.

**Figure 2:**
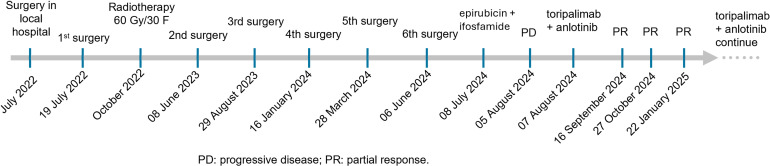
The treatment and follow-up timeline for the patient.

**Figure 3:**
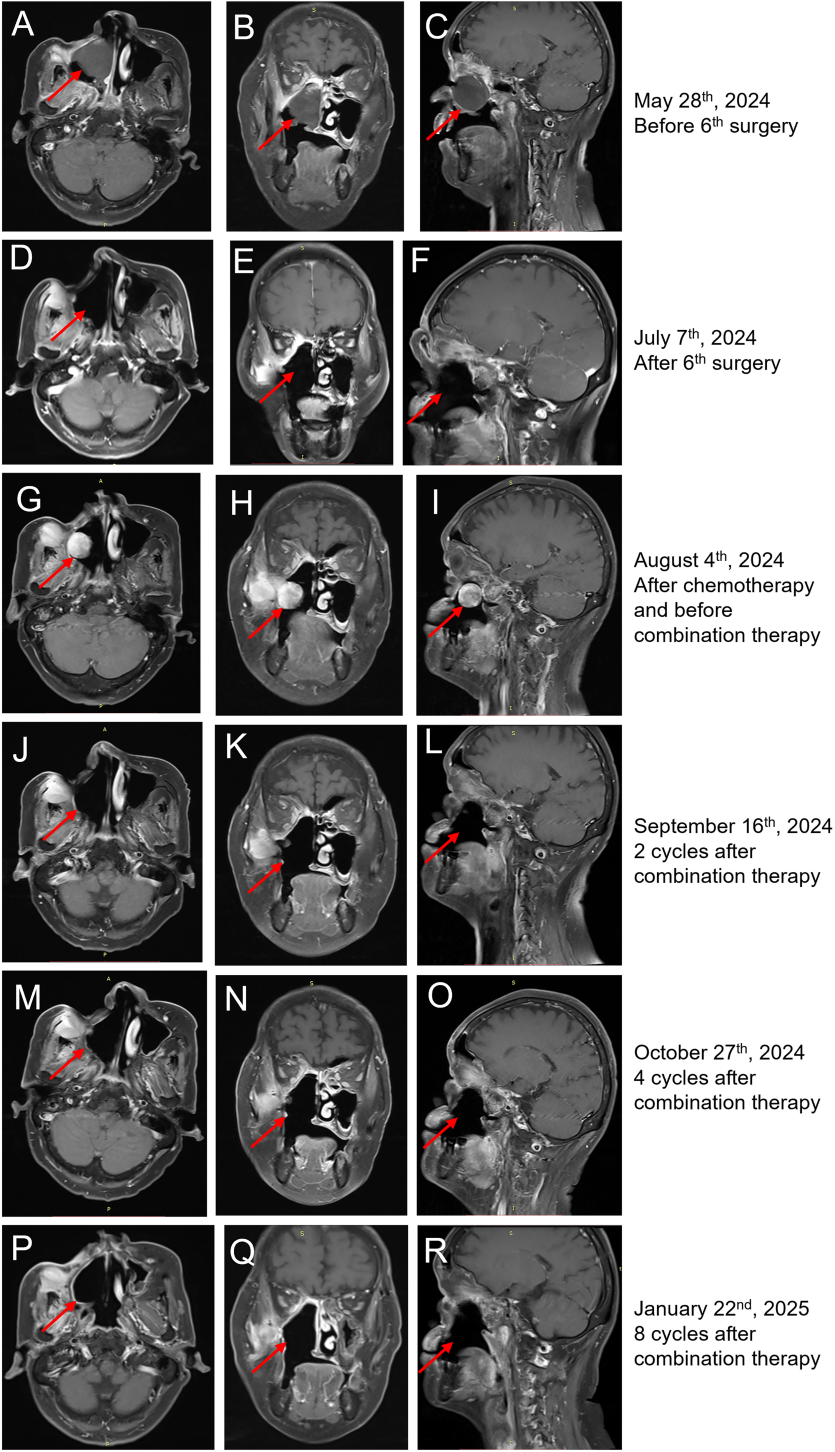
MRI findings of the case. **(A–C)** Horizontal **(A)**, coronal **(B)**, and sagittal **(C)** MRI of sinus before the sixth surgery on 28 May 2024. The tumor is indicated by red arrows. **(D–F)** Horizontal **(D)**, coronal **(E)**, and sagittal **(F)** MRI of sinus 1 month after the sixth surgery on 7 July 2024. **(G–I)** Horizontal **(G)**, coronal **(H)**, and sagittal **(I)** MRI of sinus before toripalimab + anlotinib treatment showed that the right nasal cavity, sinus, right maxillary sinus, turbinate, and palate displayed postoperative changes. The maxillofacial area was sunken, and the adjacent soft tissues were significantly enhanced. The right temporalis muscle and internal and external pterygoid muscles were thickening, and the signal was increased. There were strips of significantly enhanced shadows in the intermuscular space, which were dumbbell-shaped from the lateral wall of the upper maxillary sinus to the sinus cavity. The size is approximately 50 × 25 × 27 mm. The tumor is indicated by red arrows. **(J–L)** Horizontal **(J)**, coronal **(K)**, and sagittal **(L)** MRI of sinus after two cycles of toripalimab + anlotinib treatment showed reduced tumor lesions. **(M–O)** Horizontal **(M)**, coronal **(N)**, and sagittal **(O)** MRI of sinus after four cycles of toripalimab + anlotinib treatment showed continuous partial response. **(P–R)** Horizontal **(P)**, coronal **(Q)**, and sagittal **(R)** of sinus after eight cycles of toripalimab + anlotinib treatment showed continuous partial response.

The patient was treated with postoperative radiotherapy 60 Gy/30 F in the Department of Radiation Oncology, 3 months after the first surgery. The tumor recurred after 1 year, with recurrent lesions observed multiple times following surgeries. In detail, four more surgeries were performed by nasal endoscopy on 8 June 2023, 29 August 2023, 16 January 2024, and 28 March 2024, separately (**Figure 2**). The last surgery was performed on 6 June 2024 to remove the recurrent tumor at the right nasal sinus and further repair the wound by forehead and neck flap transfer (**Figures 2**, **3A–F**). Afterward, the patient received standard first-line chemotherapy with epirubicin + ifosfamide, which resulted in progressive disease (PD) (**Figures 2**, **3G–I**). The patient was then treated with toripalimab (240 mg, Q3W) in combination with anlotinib (orally once daily at 10 mg on days 1–14, followed by 1 week off, every 3 weeks per cycle). No significant drug-related adverse reactions were found. MRI indicated the treatment was effective after two cycles as the long diameter of the tumor was reduced by more than 50% (**Figures 3J–L**) compared with the baseline (before toripalimab + anlotinib treatment). Efficacy evaluation was PR. The patient was under this combination treatment thereafter. No grade 3 or higher adverse events (AEs) were reported during this period. Hypothyroidism was observed during a follow-up visit on 19 January 2025, with free thyroxine levels recorded at 4.300 pmol/L, below the normal range of 12.80–21.30 pmol/L. Oral administration of Euthyrox was initiated to restore the free thyroxine levels. The patient received continuous PR after two more cycles and again after four additional cycles, as confirmed by MRI scans conducted on 27 October 2024 and 22 January 2025, respectively (**Figures 3M–R**). The patient felt that the tumor remained stable during her recent follow-up visit on 24 March 2025. Thus, the combination therapy with toripalimab and anlotinib has successfully maintained tumor stasis for 7.5 months.

## Clinicopathological and genomic alterations

**Figure 4:**
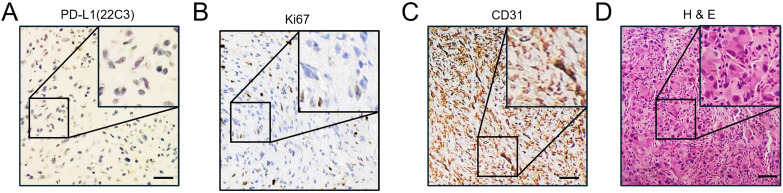
Immunohistochemical staining of CD31, Ki67, and PD-L1. **(A)** Immunohistochemical DAB staining showed negative expression of PD-L1(22C3) on sarcoma tissues before the initiation of systemic treatment. Scale bar, 50 μm. **(B)** Immunohistochemical DAB staining showed the expression of Ki67 on the sarcoma tissues before the initiation of systemic treatment. Scale bar, 50 μm. **(C)** Immunohistochemical DAB staining showed high expression of CD31 on the sarcoma tissues before the initiation of systemic treatment. Scale bar, 50 μm. **(D)** Routine hematoxylin and eosin (H&E) staining of the sarcoma tissue before the initiation of systemic treatment. Scale bar, 50 μm.

Given the significant response to toripalimab in combination with anlotinib treatment, the expression of PD-L1 and Ki67 and the establishment of blood vessels in the sarcoma tissues were investigated. Samples were collected from the last surgery before the initiation of systemic therapy, and IHC was performed (**Figure 4**). As indicated, the combined positive score (CPS) was 0 per the PD-L1(22C3) staining (**Figure 4A**). Additionally, there was a reduction in tumor proliferation, as indicated by a decrease in Ki67-positive cells from earlier tests to 20% (**Figures 1B**, **4B**). Tumor vasculature was well established as shown by CD31 positive staining (**Figure 4C**). This was not the case with the sarcoma tissue collected from the first surgery in our hospital (**Figure 1B**). The abundant vessels indicate that neovasculature was one of the factors promoting the recurrence of UPS.

Next, the lesion was evaluated for genomic alterations that may represent significant driver genes or other molecular mechanisms that could help explain the remarkable therapeutic response. The lesion and peripheral blood were screened for somatic and germline mutations, separately, using targeted next-generation sequencing (NGS) against a panel of sarcoma-associated genes, including tumor driver genes, vital tumor-related genes, drug response-related genes, tumor genetics-related genes, and immunotherapy efficacy-related genes (**Supplementary Tables 1**-**3**). The candidate genomic regions were enriched using specific probes and subsequently sequenced on the NextSeq 550 System from Illumina, achieving a read depth of over 200× (18, 19). All mutation data are summarized in **Table 1**. Notably, copy numbers (CNs) for *cyclin-dependent kinase inhibitor (CDKN) 2A* (*CDKN2A*) and *CDKN2B* in the sarcoma tissue were 0.6 and 0.5, respectively (**Table 1**). Additionally, a missense mutation of *Guanine nucleotide-binding protein G(s) subunit alpha* (*GNAS*) [NM_000516.6: c.602G>A (p.R201H)] was detected with a variant abundance of 36.93% (**Table 1**). Germline mutations including missense variants were found in genes including *Checkpoint kinase 2* (*CHEK2*), *DENN Domain Containing 1A* (*DENND1A*), *FA Complementation Group G* (*FANCG*), FA *Complementation Group I* (*FANCI*), and *Phosphodiesterase 11A* (*PDE11A*) associated with DNA damage/repair and the cell cycle (**Table 1**), although their significance is uncertain because of the absence of experimental evidence (20–24).

**Table 1:** Genomic mutation summary.

Gene symbol	Mutation site	Copy number/variant abundance	Mutation type	dbSNP
*CDKN2A*	Copy number loss	CN: 0.6	Somatic mutation	
*CDKN2B*	Copy number loss	CN: 0.5	Somatic mutation	
*GNAS*	NM_000516.6: c.602G>A (p.R201H)	36.93%	Somatic mutation	
*CHEK2*	NM_001005735.2: c.667C>T (p.R223C)	51.40%	Germline mutation	rs77130927
*DENND1A*	NM_001352964.1: c.2165_2166inv (p.S722L)	80.45%	Germline mutation	
*FANCG*	NM_004629.1: c.55A>G (p.K19E)	75.05%	Germline mutation	rs186641344
*FANCI*	NM_018193.3: c.2875C>T (p.R959W)	52.46%	Germline mutation	rs149167939
*PDE11A*	NM_001077196.2: c.700G>A (p.A234T)	50.90%	Germline mutation	rs201629965

Formalin-Fixed Paraffin-Embedded tissue (FFPE) tissues were gathered on 19 July 2022 for somatic mutation detection. Peripheral blood was collected on 24 November 2022 for germline mutation detection.

CN, copy number.

Bilateral pulmonary nodules were observed in the patient, but these remained stable with clear edges during treatment. There was no evidence that the nodules metastasized from the sinonasal tract. Lymph node metastasis was not found.

## Discussion

Malignant lesions involving the sinonasal tract account for 3% of all head and neck malignancies (25). UPS, formerly known as malignant fibrous histiocytoma (MFH) (26), makes up 25% of all sinonasal sarcomas (4). UPS of the sinonasal tract is a high-grade aggressive soft-tissue sarcoma, but due to its rarity, evidence-based therapeutic strategies are lacking. There are few reported cases of MFH/UPS, and only retrospective studies of treatment have been reported (3, 27, 28).

A retrospective review of the literature on sinonasal sarcomas from 1987 to 2017 indicated that combined modality treatment (surgery + radiation + chemotherapy) was associated with higher survival rates than single-modality therapy in sinonasal sarcoma (4). Indeed, few patients with UPS of the sinonasal region benefited from combined modality treatment (surgery + radiation + chemotherapy), resulting in a relatively long remission term (27). The 3−year overall survival (OS) rate and recurrence−free survival (RFS) rate were 59.0% and 43.5%, respectively (3). This literature is out of date and cannot offer clues to improve our treatment outcome, as the conventional treatment (surgery + radiation + chemotherapy) induced frequent recurrence and PD in our case. Thus, the treatment was changed to an immunotherapy combination with anti-angiogenesis therapy.

It was reported that patients receiving radical resection showed improved 3−year OS and PFS (79.8% and 61.9%, respectively) compared with non-radical resections (28.1% and 18.5%, respectively) (3). The primary lesion of our study patient was initially removed by non-radical resection before later extensive resection accompanying radiotherapy as adjuvant therapy. Nonetheless, the disease recurred less than 11 months post-operation, developing quickly and becoming unresectable after five more surgeries. The rapid growth characteristics of the sarcoma appear consistent with the multiple somatic mutations that were detected involving *CDNK2A*, *CDKN2B*, and *GNAS*. The CN loss of *CDNK2A* and *CDKN2B* predicts loss of CDKN2A and CDKN2B expression, uncoupling the inhibitory role on CDK4/6 to promote tumor cell proliferation (29, 30). Moreover, the missense mutation in *GNAS* would cause the accumulation of cyclic adenosine monophosphate (cAMP) by blocking the transition from guanosine-5′-triphosphate (GTP) to guanosine diphosphate (GDP). The ensuing activation of pathways downstream of cAMP would also facilitate tumor cell proliferation (31, 32).

Generally, chemotherapy works by targeting rapidly dividing cells, inducing excessive DNA damage that triggers programmed cell death responses (33). The response of UPS to chemotherapy is variable (34). However, we observed PD very soon after treatment with epirubicin + ifosfamide. Tracking the expression levels of Ki67, a marker of proliferation, showed 80% positive cells during the first surgery, which decreased to 20% during the last surgery (**Figures 1B**, **4B**). This suggests that tumor cell proliferation slowed in response to the various treatments administered, likely due to radiotherapy. Notably, the patient harbored several germline mutations in genes associated with DNA damage and DNA repair, potentially altering the effectiveness of chemotherapy by dampening DNA repair responses, leading the tumor cells to evade failsafe cell death induction mechanisms. For example, the protein product of *CHEK2* responds to DNA damage and replication blocks, playing a role as a cell cycle checkpoint regulator and a putative tumor suppressor (35). However, it remains uncertain whether the missense mutation of *CHEK2* (**Table 1**) contributed to the resistance. Further experimental and clinical data need to be collected to confirm this hypothesis.

Anlotinib was developed as an oral molecular TKI that targets vascular endothelial growth factor 1 (VEGFR1), VEGFR2, VEGFR3, platelet-derived growth factor receptors (PDGFR) α, c-Kit, and fibroblast growth factor receptors (FGFRs) 1–3 and inhibits tumor angiogenesis and tumor cell proliferation (9, 12). The ALTER0203 clinical trial showed that anlotinib had antitumor effects on advanced STS after the failure of standard chemotherapy (13, 36). Postchemotherapy maintenance treatment with anlotinib exhibits promising efficacy and tolerable toxicity in patients with advanced STS (9, 10). Anlotinib monotherapy also exhibits reasonable clinical efficacy (37). Intriguingly, IHC tracing of CD31, a biomarker of microvessel density, showed that the case sample collected from the first surgery was negative, although tissue collected from the last surgery displayed strong CD31 staining, indicating abundant neovasculature. Moreover, since CD31-labeled circulating endothelial cells serve as a predictor in anlotinib-treated non-small-cell lung cancer (38), this suggests that CD31 may be a marker for the response of UPS to anlotinib and toripalimab. However, more clinical data need to be collected to draw epidemiological conclusions.

Toripalimab is a recombinant humanized PD-1 monoclonal antibody that has been globally approved for the treatment of melanoma and nasopharyngeal carcinoma (39, 40). Toripalimab combined with doxorubicin is effective in patients with metastatic STS as a first-line treatment with manageable adverse events (41). The combination treatment with toripalimab and anlotinib showed promising efficacy and manageable safety in Chinese patients with unresectable Hepatocellular carcinoma (HCC) in the first-line setting (42, 43). Clinical trials also indicated that the maintenance therapy with toripalimab and anlotinib is a promising treatment option for patients with advanced STS after first-line anthracycline-based chemotherapy (44). One UPS patient reported to be non-responsive to anlotinib monotherapy benefited from subsequent anlotinib–toripalimab combination therapy over 23 months (17). Our patient case was PD-L1 negative, although recent evidence suggests that even PD-L1-negative tumors may respond to PD-1 inhibitors, possibly due to other immune-related mechanisms (45, 46). Indeed, it has been reported that a combination of anti-angiogenic therapy and immune checkpoint blockade normalizes vascular-immune crosstalk to potentiate cancer immunity (47). Considering the desperate disease progression, we directly chose combined modality treatment with toripalimab and anlotinib with a significant PR recorded after just two treatment cycles. The efficacy of this combination therapy rekindled the hopes of the patient, and PR was achieved after four cycles and again after eight cycles. As the patient could feel changes in the size of the sarcoma, the patient reported that it remained stable after the 11th cycle was completed. To avoid excessive imaging, no further MRI was conducted until the submission of our manuscript. The patient was disappointed with the outcome of surgeries and chemotherapy treatment but continued to diligently follow the advice of her treating physician. The patient remains optimistic that the disease could be controlled with this combination treatment as long as possible.

Exploring the changes in biological behaviors in sarcoma tissues after the combination treatment would be helpful for understanding the underlying mechanisms. However, the patient refused any further tissue collection for biopsy, which, given her extensive surgical history, is understandable.

## Conclusion

This case provides therapeutic confidence in the treatment of sinonasal UPS, with a view to the further application of toripalimab plus anlotinib. Given the difficulties in implementing large clinical trials, further respective studies are now needed to confirm the benefits of the toripalimab and anlotinib combination treatment.

## Data Availability

The original contributions presented in the study are included in the article/**Supplementary Material**. Further inquiries can be directed to the corresponding author.
