# Supplementary material for: Efficacy of toripalimab in combination with anlotinib in recurrent undifferentiated pleomorphic sarcoma of the sinonasal region: a case report with biomarker analysis
Source: Front Immunol. 2025 May 9;16:1541209. doi: 10.3389/fimmu.2025.1541209 (PMC12098334; doi:10.3389/fimmu.2025.1541209)
Supplement: Supplementary file 1 [file Table1.docx]

**Table S1. The 481 listed genes that were analysis with single-nucleotide polymorphism (SNP), insertion and deletion (indel).**

| *ABCB1(MDR1)* | *ABCC2(MRP2)* | *ADGRB3(BAI3)* | *ADH1B* | *AFDN(MLLT4)* |
| --- | --- | --- | --- | --- |
| *AIP* | *AKT1* | *AKT2* | *AKT3* | *ALDH2* |
| *ALK* | *AMER1(FAM123B)* | *APC* | *AR* | *ARAF* |
| *ARID1A* | *ARID1B* | *ARID2* | *ARID5B* | *ASCL4* |
| *ASXL1* | *ATF1* | *ATIC* | *ATM* | *ATR* |
| *ATRX* | *AURKA* | *AURKB* | *AXIN2* | *AXL* |
| *B2M* | *BAD* | *BAK1* | *BAP1* | *BARD1* |
| *BAX* | *BCL2* | *BCL2L11(BIM)* | *BCOR* | *BCORL1* |
| *BCR* | *BIRC3* | *BLM* | *BMPR1A* | *BRAF* |
| *BRCA1* | *BRCA2* | *BRD4* | *BRIP1* | *BTG2* |
| *BTK* | *BUB1B* | *CASP8* | *CBL* | *CBLB* |
| *CCN6(WISP3)* | *CCND1* | *CCNE1* | *CD274(PD-L1)* | *CD74* |
| *CDA* | *CDC73* | *CDH1* | *CDK10* | *CDK12* |
| *CDK4* | *CDK6* | *CDK8* | *CDKN1A* | *CDKN1B* |
| *CDKN1C* | *CDKN2A* | *CDKN2B* | *CDKN2C* | *CEBPA* |
| *CEP57* | *CHD4* | *CHD8* | *CHEK1* | *CHEK2* |
| *CIC* | *CITED2* | *COL1A1* | *CREB1* | *CREBBP* |
| *CRKL* | *CSF1R* | *CTCF* | *CTLA4* | *CTNNB1* |
| *CUL3* | *CUX1* | *CXCR4* | *CYLD* | *CYP19A1* |
| *CYP2A13* | *CYP2A6* | *CYP2A7* | *CYP2B6* | *CYP2C19* |
| *CYP2C9* | *CYP2D6* | *CYP3A4* | *CYP3A5* | *CYSLTR2* |
| *DAXX* | *DDIT3* | *DDR2* | *DENND1A* | *DHFR* |
| *DICER1* | *DLL3* | *DNMT3A* | *DOT1L* | *DPYD* |
| *DTL(CDT2)* | *DUSP2* | *EED* | *EGFR* | *EIF1AX* |
| *EML4* | *EMSY(c11orf30)* | *EP300* | *EPAS1* | *EPC1* |
| *EPCAM* | *EPHA2* | *EPHA3* | *EPHA5* | *ERBB2(HER2)* |
| *ERBB3* | *ERBB4* | *ERBIN(ERBB2IP)* | *ERCC1* | *ERCC2* |
| *ERCC3* | *ERCC4* | *ERCC5* | *ERG* | *ESR1* |
| *ETV1* | *ETV4* | *ETV5* | *ETV6* | *EWSR1* |
| *EXT1* | *EXT2* | *EZH2* | *EZR* | *FANCA* |
| *FANCC* | *FANCD2* | *FANCE* | *FANCF* | *FANCG* |
| *FANCI* | *FANCL* | *FANCM* | *FAT1* | *FBXW7* |
| *FEV* | *FGF19* | *FGFR1* | *FGFR2* | *FGFR3* |
| *FGFR4* | *FH* | *FLCN* | *FLT1(VEGFR1)* | *FLT3* |
| *FLT4* | *FOXA1* | *FOXL2* | *FOXO1* | *FOXO4* |
| *FOXP1* | *FRG1* | *FUS* | *GATA1* | *GATA2* |
| *GATA3* | *GATA4* | *GATA6* | *GLI1* | *GNA11* |
| *GNAQ* | *GNAS* | *GREB1* | *GREM1* | *GRIN2A* |
| *GRM3* | *GRM8* | *GSTM1* | *GSTM4* | *GSTP1* |
| *GSTT1* | *H3-3A(H3F3A)* | *H3-3B(H3F3B)* | *HDAC2* | *HDAC9* |
| *HEY1* | *HGF* | *HLA-A* | *HMGA2* | *HNF1A* |
| *HNF1B* | *HOXB13* | *HRAS* | *IDH1* | *IDH2* |
| *IFNA6* | *IFNB1* | *IFNE* | *IFNG* | *IFNGR1* |
| *IFNGR2* | *IGF1R* | *IGF2* | *IKBKE* | *IKZF1* |
| *IL7R* | *INPP4B* | *IRF2* | *IRX2* | *JAK1* |
| *JAK2* | *JAK3* | *JARID2* | *JUN* | *KDM5A* |
| *KDR(VEGFR2)* | *KEAP1* | *KIF1B* | *KIT* | *KITLG* |
| *KLLN* | *KMT2A(MLL)* | *KMT2B* | *KMT2C* | *KMT2D(MLL2)* |
| *KRAS* | *LHCGR* | *LMO1* | *LRP1* | *LRP1B* |
| *LYN* | *LZTR1* | *MAP2K1(MEK1)* | *MAP2K2(MEK2)* | *MAP2K4* |
| *MAP3K1* | *MAP3K4* | *MAX* | *MC1R* | *MCL1* |
| *MDM2* | *MDM4* | *MECOM* | *MED12* | *MEF2B* |
| *MEN1* | *MET* | *MGMT* | *MITF* | *MLH1* |
| *MLH3* | *MLLT1* | *MLLT3* | *MPL* | *MRE11(MRE11A)* |
| *MSH2* | *MSH6* | *MTAP* | *MTHFR* | *MTOR* |
| *MUTYH* | *MYH9* | *MYBL1* | *MYC* | *MYCL(MYCL1)* |
| *MYCN* | *MYD88* | *NAB2* | *NAT1* | *NBN* |
| *NCOA2* | *NCOR1* | *NF1* | *NF2* | *NFATC2* |
| *NFE2L2* | *NFKBIA* | *NKX2-1* | *NOTCH1* | *NOTCH2* |
| *NOTCH3* | *NPM1* | *NQO1* | *NR4A3* | *NRAS* |
| *NRG1* | *NSD1* | *NTHL1* | *NTRK1* | *NTRK2* |
| *NTRK3* | *NUTM1* | *NUTM2A* | *NUTM2B* | *PAK3* |
| *PALB2* | *PALLD* | *PARP1* | *PARP2* | *PATZ1* |
| *PAX3* | *PAX5* | *PAX7* | *PBRM1* | *PDCD1(PD1)* |
| *PDCD1LG2(PD-L2)* | *PDE11A* | *PDGFB* | *PDGFRA* | *PDGFRB* |
| *PDK1* | *PGR* | *PHF1* | *PHOX2B* | *PIK3C3* |
| *PIK3CA* | *PIK3CD* | *PIK3R1* | *PIK3R2* | *PKHD1* |
| *PLAG1* | *PLCB4* | *PLK1* | *PMS1* | *PMS2* |
| *POLD1* | *POLD3* | *POLE* | *POLH* | *POT1* |
| *PPARD* | *PPM1D* | *PPP2R1A* | *PPP2R2A* | *PRDM1* |
| *PREX2* | *PRF1* | *PRKACA* | *PRKAR1A* | *PRKCI* |
| *PRKDC* | *PRKN(PARK2)* | *PRSS1* | *PRSS3* | *PTCH1* |
| *PTEN* | *PTK2* | *PTPN11* | *PTPN13* | *QKI* |
| *RAC1* | *RAC3* | *RAD50* | *RAD51* | *RAD51B* |
| *RAD51C* | *RAD51D* | *RAD54L* | *RAF1* | *RARA* |
| *RARG* | *RASGEF1A* | *RB1* | *RECQL4* | *RELA* |
| *RELN* | *RET* | *RHBDF2* | *RHOA* | *RICTOR* |
| *RNF43* | *ROS1* | *RPTOR* | *RRM1* | *RUNX1* |
| *RUNX1T1* | *SBDS* | *SDC4* | *SDHA* | *SDHB* |
| *SDHC* | *SDHD* | *SEPTIN9(SEPT9)* | *SETBP1* | *SETD2* |
| *SF3B1* | *SGK1* | *SKP2* | *SLC34A2* | *SLC3A2* |
| *SMAD2* | *SMAD3* | *SMAD4* | *SMAD7* | *SMARCA4* |
| *SMARCB1* | *SMO* | *SOCS1* | *SOS1* | *SOX2* |
| *SPOP* | *SPRED1* | *SPRY4* | *SRC* | *SRF* |
| *SRSF2* | *SRY* | *SS18* | *SSX1* | *SSX2* |
| *SSX4* | *STAG2* | *STAT3* | *STK11* | *STMN1* |
| *SUFU* | *SUZ12* | *TACC3* | *TAF15* | *TAP1* |
| *TAP2* | *TEK* | *TEKT4* | *TERC* | *TERT* |
| *TET2* | *TFE3* | *TGFBR2* | *THADA* | *TMEM127* |
| *TMPRSS2* | *TNFAIP3* | *TNFRSF11A* | *TNFRSF14* | *TNFRSF19* |
| *TNFSF11* | *TOP1* | *TOP2A* | *TP53* | *TP63* |
| *TPMT* | *TSC1* | *TSC2* | *TSHR* | *TSPAN31* |
| *TTF1* | *TUBB3* | *TYMS* | *U2AF1* | *WT1* |
| *UGT1A1* | *VAMP2* | *VEGFA* | *VGLL2* | *VHL* |
| *WAS* | *WRN* | *XPA* | *XPC* | *XRCC1* |
| *XRCC2* | *YAP1* | *YWHAE* | *ZC3H7B* | *ZNF217* |
| *ZNF703* |  |  |  |  |

**Table S2. The 104 listed genes that were analysis with gene rearrangement.**

| *AKT2* | *ALK* | *ATF1* | *AXL* | *BCL2* |
| --- | --- | --- | --- | --- |
| *BCOR* | *BCORL1* | *BCR* | *BIRC3* | *BRAF* |
| *BRCA1* | *BRCA2* | *BRD4* | *CCND1* | *CD274(PD-L1)* |
| *CD74* | *CDK12* | *CDK4* | *CDKN2A* | *CIC* |
| *CITED2* | *COL1A1* | *CREB1* | *DDIT3* | *EGFR* |
| *EML4* | *EPC1* | *ERBB2(HER2)* | *ERBB3* | *ERG* |
| *ETV1* | *ETV4* | *ETV6* | *EWSR1* | *EZR* |
| *FEV* | *FGFR1* | *FGFR2* | *FGFR3* | *FOXO1* |
| *FOXO4* | *FUS* | *GLI1* | *GREB1* | *H3F3A* |
| *H3F3B* | *HEY1* | *IRX2* | *JAK2* | *KIT* |
| *KMT2A(MLL)* | *LRP1* | *MET* | *MTAP* | *MYBL1* |
| *MYC* | *NAB2* | *NCOA2* | *NFATC2* | *NOTCH1* |
| *NOTCH2* | *NPM1* | *NR4A3* | *NRG1* | *NTRK1* |
| *NTRK2* | *NTRK3* | *NUTM1* | *NUTM2A* | *NUTM2B* |
| *PATZ1* | *PAX3* | *PAX7* | *PDGFB* | *PDGFRA* |
| *PDGFRB* | *PHF1* | *PRKACA* | *RAD50* | *RAF1* |
| *RARA* | *RELA* | *RELN* | *RET* | *ROS1* |
| *RUNX1* | *RUNX1T1* | *SDC4* | *SLC34A2* | *SRF* |
| *SS18* | *SSX1* | *SSX2* | *SSX4* | *TACC3* |
| *TAF15* | *TFE3* | *TMPRSS2* | *TP53* | *VGLL2* |
| *WT1* | *YAP1* | *YWHAE* | *ZC3H7B* |  |

Table S3. The 106 listed genes that were analysis with copy number variation (CNV).

| *ABCB1(MDR1)* | *AKT1* | *AKT2* | *AKT3* | *ALK* |
| --- | --- | --- | --- | --- |
| *APC* | *AR* | *AURKB* | *AXL* | *BCL2* |
| *BMPR1A* | *BRAF* | *CCND1* | *CCNE1* | *CD274(PD-L1)* |
| *CDH1* | *CDK12* | *CDK4* | *CDK6* | *CDKN2A* |
| *CDKN2B* | *CRKL* | *CSF1R* | *DDR2* | *DLL3* |
| *EGFR* | *EPCAM* | *ERBB2(HER2)* | *ERBB3* | *ESR1* |
| *FGF19* | *FGFR1* | *FGFR2* | *FGFR3* | *FGFR4* |
| *FLT1(VEGFR1)* | *FLT3* | *FLT4* | *GLI1* | *GNAS* |
| *HGF* | *HMGA2* | *HRAS* | *IGF1R* | *IGF2* |
| *IL7R* | *JAK1* | *KIT* | *KRAS* | *MAP2K1(MEK1)* |
| *MAP2K2(MEK2)* | *MCL1* | *MDM2* | *MDM4* | *MET* |
| *MGMT* | *MLH1* | *MSH2* | *MSH6* | *MTOR* |
| *MUTYH* | *MYC* | *MYCN* | *NF1* | *NKX2-1* |
| *NOTCH2* | *NRAS* | *NTRK1* | *PDCD1LG2(PD-L2)* | *PDGFRA* |
| *PDGFRB* | *PIK3CA* | *PMS2* | *POLD1* | *POLE* |
| *PTEN* | *PTK2* | *RB1* | *RET* | *RICTOR* |
| *RRM1* | *SDHA* | *SDHB* | *SDHC* | *SDHD* |
| *SMAD4* | *SMARCB1* | *SMO* | *SOX2* | *SRC* |
| *STK11* | *STMN1* | *TERC* | *TERT* | *TOP1* |
| *TOP2A* | *TP53* | *TSC1* | *TSC2* | *TSPAN31* |
| *TTF1* | *TUBB3* | *VEGFA* | *VHL* | *ZNF217* |
| *ZNF703* |  |  |  |  |
